# Letter to the editor: multiple introductions of MPX in Italy from different geographic areas

**DOI:** 10.2807/1560-7917.ES.2022.27.23.2200456

**Published:** 2022-06-09

**Authors:** Federica Ferraro, Anna Caraglia, Alessia Rapiti, Danilo Cereda, Francesco Vairo, Giovanna Mattei, Francesco Maraglino, Giovanni Rezza

**Affiliations:** 1 Directorate General of Health Prevention, Ministry of Health, Italy; 2 Directorate General for Health, Lombardy Region, Milano, Italy; 3 National Institute of Infectious Diseases Lazzaro Spallanzani, Italy; 4 General Directorate for personal care, health and welfare, Emilia Romagna Region, Bologna, Italy

**Keywords:** monkeypox, Italy, rash, surveillance

**To the editor:** In last week’s issue of *Eurosurveillance*, Antinori et al. [1] comprehensively described the first four cases of monkeypox detected in Rome, all of them in men who have sex with men (MSM) presenting with ano-genital rash, and who had travelled to other European countries. Here we share some considerations on the need to also take into account the possibility of monkeypox virus (MPXV) introduction from non-European countries during the course of the current outbreak, as suggested by the epidemiological investigations conducted in Italy on further confirmed recent monkeypox cases.

Following the first reports of autochthonous cases of MPX in several European countries at the beginning of May, the World Health Organization (WHO) and the European Centre for Disease Prevention and Control alerted member states to report suspected and/or confirmed cases [2,3]. Apart from early cases reported in the United Kingdom (UK), one of whom had travelled to Nigeria [4], cases were mostly identified in adult males, and in particular in MSM [4,5]. Retrospective investigations in Portugal and in the UK indicate symptom onset of the first cases dated back at least to April 2022 [5,6]. Although the analysis of available virus sequences from the present outbreak strongly suggests that cases in the different European countries are caused by the West African clade of the MPXV, none of the cases reported travels to Nigeria or other Western African countries [4,6].

As at 6 June 2022, a total number of 29 PCR-confirmed cases matching the criteria of the WHO case definition were reported in Italy (Figure). All except one were males, and 16 of 18 reported having sex with other men; the median age of cases was 36 years (range: 20–54 years). All presented with a rash; in 18 of 21 cases, the rash was localised in the genital/perianal area; information on the distribution of the lesions was missing for remaining cases. Fever was reported in 12 of 22 cases for whom information was available.

**Figure:**
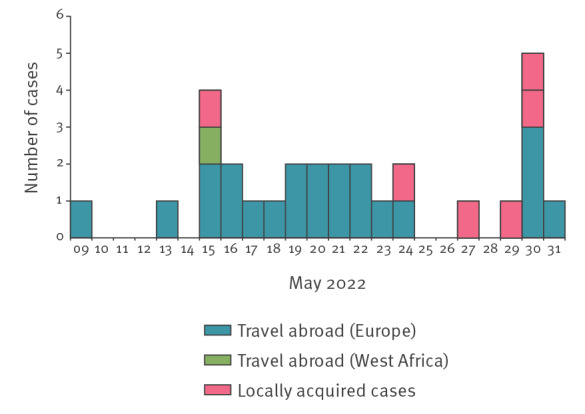
Confirmed cases of monkeypox by date of onset of symptoms and exposure, Italy, data as at 6 June 2022 (n = 29)

Overall, 23 of 29 cases had travelled abroad, and most of them (13/23) had spent a vacation period on the Canary Islands, suggesting the occurrence of a major amplifying event. In locally acquired cases, the epidemiological investigation revealed a chain of transmission of two generations of locally acquired cases related to a confirmed index case returning to Italy from Ghana. To this regard, it is worth mentioning that the MPX outbreak that occurred in the United States in 2003 was initiated by direct contact with a rodent imported from Ghana [7].

Following the identification of the current multi-country outbreak, the Italian Ministry of Health issued a series of recommendations, including case notification, protective measures (contact and droplet) for healthcare workers, contact tracing with self-surveillance of close contacts, and the possibility of implementing quarantine measures to be evaluated by local health authorities in particular epidemiological and/or environmental contexts.

We would like to stress here that most cases identified in Italy so far are not locally acquired, and the link with different geographic areas (i.e., Europe and West Africa) underlines the possibility of multiple independent introductions of the virus, as well as the possibility of a wider spread in the areas where the disease is endemic. Although the number of cases is still limited, it is important to strengthen surveillance and control activities, from early case detection and notification to contact tracing. Furthermore, maintaining a high level of public attention, providing non-stigmatising information to at-risk population groups, is also key in order to contain the spread of MPX virus, also considering the seasonal intensity of aggregation events and recreational activities.
